# Supplementary material for: The effect of ergometer cycling and visual foraging on brain function: A pilot study
Source: PLoS One. 2025 Dec 1;20(12):e0336642. doi: 10.1371/journal.pone.0336642 (PMC12668562; doi:10.1371/journal.pone.0336642)
Supplement: Attachment — Submitted filename: Response Document 2.docx [file pone.0336642.s004.docx]

Response Document 2

**Reviewer #1: The authors have addressed most of the comments raised previously. Although I do not fully agree with all of the responses and additional explanations, the revised manuscript is generally convincing. However, the issue of the power analysis still remains.**

**First, I agree that a sample size of 27 is adequate for the current study. Nevertheless, as noted in my previous comment, G*Power cannot directly perform a power analysis for a two-way repeated measures ANOVA model. Therefore, I assume that the authors conducted the analysis using either “ANOVA: Repeated measures, within factors,” “ANOVA: Repeated measures, within–between interaction,” or another approximation. The current explanation does not provide sufficient detail for readers to reproduce the analysis. I encourage the authors to clearly specify the exact procedure they used in G*Power.**

Thank you for your helpful comment regarding the sample size estimation – and please accept our apologies for not fully addressing your comment first time around. We have now explicitly explained the power analysis procedure in the revised manuscript. This information has been added to the Methods section (Lines 116-130) to increase transparency and reproducibility:

“Sample size was estimated using G*Power 3.1 (35). Estimates were based on a published effect size of *d* = 0.52 for acute moderate intensity cycling on EF task performance (3), experimental power of 0.80, and a significance level of 0.05 for a repeated measures ANOVA (Condition [EC, VF, EC+VF] x Time [pre-, post-intervention), to yield a desired sample size of 17 participants. However, G*Power does not derive estimates for interaction effects for mixed-design repeated measures ANOVAs, this figure may represent an underestimation. Further, because this was a pilot study – one that required multiple demanding laboratory visits from participants leading to potential participant attrition - a total of 27 participants were recruited. This sample size is also comparable to those used in previous studies examining the effects of dual-tasking (17,36–38).

We adopted the ‘ANOVA: Repeated measures, within factors’ G*Power analysis protocol to approximate the sample size for a within-subjects design. The parameters were as follows: an effect size of Cohen’s *f* = 0.26 (converted from Cohen’s d = 0.52), α = 0.05, power = 0.80, one group, and six repeated measurements (derived from 3 conditions x 2 time points). This analysis yielded a required sample size of 17 participants. However, to account for potential underestimation, participant attrition, and comparability with previous studies, we aimed to recruit 27 participants.”
